# GluN2B influences the progression of status epilepticus by modulating calcium ion homeostasis through its interaction with CaMKIIα

**DOI:** 10.3389/fphar.2025.1550879

**Published:** 2025-04-22

**Authors:** Lin Zhang, Youshi Meng, Chaoning Liu, Lei Wei, Yuling Lu, Shouhuan Zheng, Donghua Zou, Yuan Wu

**Affiliations:** ^1^ Department of Neurology, The First Affiliated Hospital of Guangxi Medical University, Nanning, Guangxi, China; ^2^ Department of Neurology, The Second Affiliated Hospital of Guangxi Medical University, Nanning, Guangxi, China

**Keywords:** status epilepticus, Camkiiα, phosphorylation, GluN2B, calcium homeostasis, ifenprodil

## Abstract

**Background:**

Status epilepticus (SE) is a neurological emergency characterized by prolonged, unresolved epileptic seizures, often resulting in adverse outcomes. Conventional pharmaceuticals are not universally effective in terminating epileptic seizures; therefore, identifying novel targets for seizure cessation and the prevention of SE is crucial. This study aimed to assess the expression levels and interactions of the N-methyl-D-aspartate receptor (NMDAR) subunit GluN2B and CaMKIIα following epileptic convulsions and to explore their potential mechanisms of action.

**Methods:**

This study utilized Western blotting to evaluate the protein expression levels of CaMKIIα, p-CaMKIIα, and GluN2B in the hippocampus of mice subjected to kainic acid-induced SE. Immunofluorescence colocalization analysis and co-immunoprecipitation were utilized to investigate the interaction between GluN2B and CaMKIIα in the hippocampus. Additionally, flow cytometry was employed to measure intracellular calcium ion levels.

**Results:**

Compared to the sham operation group, the intracellular calcium ion concentration in the hippocampus of SE mice was elevated, whereas the expression of p-CaMKIIα was markedly reduced. The levels of CaMKIIα and GluN2B remained unchanged, and the immune complex of GluN2B and CaMKIIα in the SE group exhibited a significant increase. The GluN2B inhibitor ifenprodil was found to prolong the latency of epileptic seizures, counteract calcium influx, and modulate the expression of p-CaMKIIα, as well as the immune complex levels of GluN2B and CaMKIIα. These findings suggest that the interaction between GluN2B and CaMKIIα may be critical in the pathophysiological processes of SE, influencing the levels of p-CaMKIIα and calcium ion homeostasis.

**Conclusion:**

The reduction in CaMKIIα phosphorylation levels depends on the NMDAR pathway. When GluN2B binds to CaMKIIα, it may occupy the autophosphorylation site of CaMKIIα (T286 binding site), thereby affecting its autophosphorylation. This results in decreased phosphorylation levels, disruption of NMDAR-dependent calcium homeostasis, and alteration of the excitation/inhibition balance.

## Graphical Abstract

**Figure:**
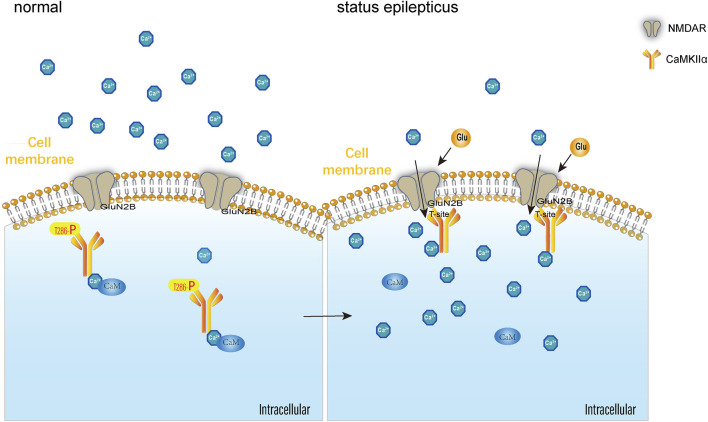
After GluN2B binds with CaMKIIa, GluN2B occupies the CaMKIIa autophosphorylation site at threonine 286 (T286), affecting CaMKIIa autophosphorylation, reducing phosphorylation levels, and disrupting NMDAR-dependent calcium ion homeostasis, thereby disrupting the excitation-inhibition balance in the central nervous system and influencing the onset and progression of SE.

## 1 Introduction

Epileptic seizures are believed to be associated with heightened neuronal excitation, diminished inhibitory function, or both, a pathological alteration known as excitation/inhibition (E/I) imbalance (Li et al., 2022; Betjemann and Lowenstein, 2015). Enhanced neuronal excitation is primarily associated with elevated glutamate (Glu) release from the presynaptic membrane and an increase in Glu receptors on the postsynaptic membrane (Deng et al., 2013; Wasterlain et al., 2013). In contrast, diminished neuronal inhibitory function correlates with reduced presynaptic γ-aminobutyric acid (GABA) release and a reduction in postsynaptic GABA receptors (Wasterlain et al., 1993; Li et al., 2023). Transient E/I imbalance may not result in significant consequences; however, prolonged E/I imbalance may precipitate status epilepticus (SE), a neurological emergency that often leads to adverse outcomes (Kapur and Coulter, 1995). Timely rectification of E/I imbalance can effectively halt SE and provide benefits for patients (Moshé et al., 2015).

Benzodiazepines, such as diazepam and lorazepam, are commonly employed in clinical practice to terminate SE (Jones-Davis and Macdonald, 2003). Unfortunately, approximately 11%–57% of patients with SE demonstrate resistance to benzodiazepines, a condition classified as refractory SE (Alldredge et al., 2001). The primary etiology of refractory SE is the persistent internalization of GABA A receptors (GABAARs) on neuronal surfaces, which serve as active sites for benzodiazepines (Kittler et al., 2002; Rudolph et al., 2001), resulting in the ineffectiveness of these medications (Mayer et al., 2002). Consequently, identifying novel therapeutic targets for SE has become a significant challenge in current research.

Calcium ions are considered the primary mediators of excitotoxic neuronal injury. Calcium influx plays a crucial role in various physiological processes. Research suggests that disturbances in calcium ion homeostasis are closely associated with the onset and progression of epilepsy (Blair et al., 2008). Calcium ion influx initiates transcription via calcium-regulated signaling pathways. Calcium/calmodulin-dependent protein kinase II (CaMKII) is the most abundant protein kinase in the brain. It consists of 6–12 subunits, designated α, β, γ, and δ, and accounts for 2% of the total protein in the hippocampus (Erondu and Kennedy, 1985; Yamagata et al., 2006). It is a major Ca^2+^-regulated signal transduction pathway in the central nervous system, functioning at both presynaptic and postsynaptic sites (Ghosh and Greenberg, 1995; Malenka and Nicoll, 1999; Churn, 1995). The activation of CaMKII, particularly the α subunit in the hippocampus, plays a crucial role in cytoskeletal remodeling, neurotransmitter synthesis and release, ion channel function, and processes related to learning and memory (Giese et al., 1998). CaMKⅡα is considered essential in the treatment of epilepsy. However, the specific role of CaMKIIα in SE and its underlying mechanisms remain unclear.

The N-methyl-D-aspartate receptor (NMDAR) is an ion channel protein and a heteromer composed of three subunits (GluN1–GluN3) (Elhussiny et al., 2021). GluN2 acts as the regulatory subunit of the NMDAR, consisting of four subtypes (GluN2A–GluN2D), and primarily functions in a modulatory role. Various subtypes of GluN2B confer distinct physiological and pharmacological properties to NMDAR channels, such as receptor channel opening duration, synaptic plasticity, Zn^2+^ sensitivity, and Ca^2+^ permeability (Markus et al., 2022). Current research primarily focuses on GluN2A and GluN2B, as these are the most prevalent GluN2 subunits. Additionally, NMDAR channels containing GluN2A and GluN2B subunits exhibit a high opening frequency, making NMDARs with these subunits a critical component of the functional NMDAR ion channel (Mahmoud et al., 2020). NMDARs are permeable to Ca^2+^, and the influx of Ca^2+^ into neurons following NMDAR activation contributes to intracellular signaling pathways (Myers et al., 2019). The hyperactivity of NMDARs, including GluN2B, may be associated with the onset of epilepsy.

Research suggests that inhibitory phosphorylation of CaMKIIα is critical for NMDAR-dependent long-term synaptic depression (Chen et al., 2020). Moreover, intense synaptic stimulation leads to the sustained incorporation of CaMKII into synapses through GluN2B binding, facilitating prolonged autonomous activity in the postsynaptic density, which may significantly contribute to synaptic plasticity (Bayer et al., 2006). Therefore, we hypothesized that GluN2B may play a role in epileptic seizures through its influence on CaMKIIα. Using an *in vivo* mouse model of SE, this study aimed to assess the expression levels and interactions of GluN2B and CaMKIIα following epileptic convulsions and to explore their potential mechanisms of action.

## 2 Materials and methods

### 2.1 Culture of primary neuronal cells

The cultivation of neuronal cells was based on the method described by Li et al. (2023).

### 2.2 Immunofluorescence colocalization analysis

Cell immunofluorescence staining followed the protocol described by Li et al. (2023). The primary antibodies used were anti-GluN2B antibody (1:200, Proteintech, 21920-1-AP) and anti-CaMKII alpha antibody (Abmart, T57228).

For immunofluorescence staining of tissue sections, paraffin-embedded mouse brain slices were dewaxed using xylene (A509900-0500, Sangon Bioengineering Co., Ltd.) and rinsed with 100% ethanol (A500737-0500, Sangon Bioengineering Co., Ltd.). Subsequently, the sections were placed in a repair box containing Ethylene Diamine Tetraacetic Acid antigen retrieval buffer (pH, 8.0) and underwent antigen retrieval in a microwave oven. Autofluorescence was quenched, and bovine serum albumin (BSA) (E675007-0100, BBI) was incrementally added for blocking. Anti-GluN2B antibody at a 1:200 dilution (Proteintech, 21920-1-AP) was applied, and the sections were incubated overnight at 4°C. The sections were further incubated with a secondary antibody at 37°C for 50 min. Anti-CaMKII antibody (Abmart, T57228) was applied and incubated overnight at 4°C, and secondary antibodies were applied at 37°C for 50 min. Finally, DAPI staining solution (E607303-0002, BBI) was added, and the sections were incubated at room temperature for 10 min. Neurons and hippocampal tissue were examined using the OLYMPUS BX53 fluorescent microscope, and micrographs were captured. Three fields of view were randomly selected from glass coverslips. The imaging parameters for the cells were as follows: microscope model: Olympus BX53; objective lens type: neuron; exposure duration: 50 ms; and optical/digital magnification: ×60. Various areas of the hippocampal tissue, including CA1, CA3, DG zone were photographed. The imaging parameters for the hippocampal tissue were as follows: microscope model: Olympus BX53; objective lens type: hippocampal tissue; exposure duration: 250 ms; and optical/digital magnification: ×200. Images were analyzed using ImageJ software, and the Pearson correlation coefficient (PCC) was calculated (Image-Color-Segmentation Channel, Image-Color-Channel Tools, Analysis-Colocalization-Coloc2). The PCC was used to determine whether there is a co-localization relationship between the proteins. PCC ranges from 0 to 1, where 0 indicates non-overlapping images and 1 indicates complete colocalization between the two images.

### 2.3 Animals

Sixty adult male *C57BL/6J* mice (6–8 weeks old, 18–22 g) were used in the experiment and sourced from Beijing Sibeifu Biotechnology Co., Ltd., China. The mice were housed under the following conditions: temperature 22°C–26°C, 50%–60% humidity, and a 12:12 light-dark cycle beginning at 08:00, with six mice per enclosure. The mice had unrestricted access to food and water. Animal care and handling were conducted in accordance with the guidelines established by the Experimental Animal Care Committee of Guangxi Medical University. The research protocol was approved by the Experimental Animal Ethics Committee of Guangxi Medical University and adhered to the principles of the Declaration of Helsinki. Pre-experimental data and statistical analyses (the Resource Equation Approach) were used to determine the minimum sample size required to reduce the number of animals while maximizing the comparative validity of the experimental results. At the end of the experiment, the animals were deeply sedated by isoflurane and euthanized to alleviate suffering.

### 2.4 Drug administration protocol

Kainic acid (KA) was obtained from MedChemExpress (MCE, USA) (catalog number: HY-N2309). Ifenprodil hemitartrate was obtained from Good Laboratory Practice Bioscience (GLPBIO), USA (catalog number: GC10471). KA was dissolved in normal saline, whereas Ifenprodil was dissolved in phosphate-buffered saline (PBS; pH, 4.2–4.5) supplemented with 6% dimethyl sulfoxide (DMSO). Mice in the sham surgery group were administered an equivalent volume of normal saline in the hippocampus. In the Ifenprodil intervention study, Ifenprodil was administered intraperitoneally for 7 days at doses of 6, 15, and 25 mg/kg, followed by stereotaxic injection of KA into the hippocampus to induce the model. In the solvent group, 1 mL of a PBS + DMSO mixture was administered intraperitoneally for 7 days before establishing the KA model. The sham operation group and the SE group received 1 mL of normal saline intraperitoneally, followed by stereotaxic injection of 1 µL of normal saline into the hippocampus of the sham operation group and 1 µL of KA into the hippocampus of the SE group.

### 2.5 Experimental design

#### 2.5.1 KA induces SE *in vivo*

**FIGURE 1:**
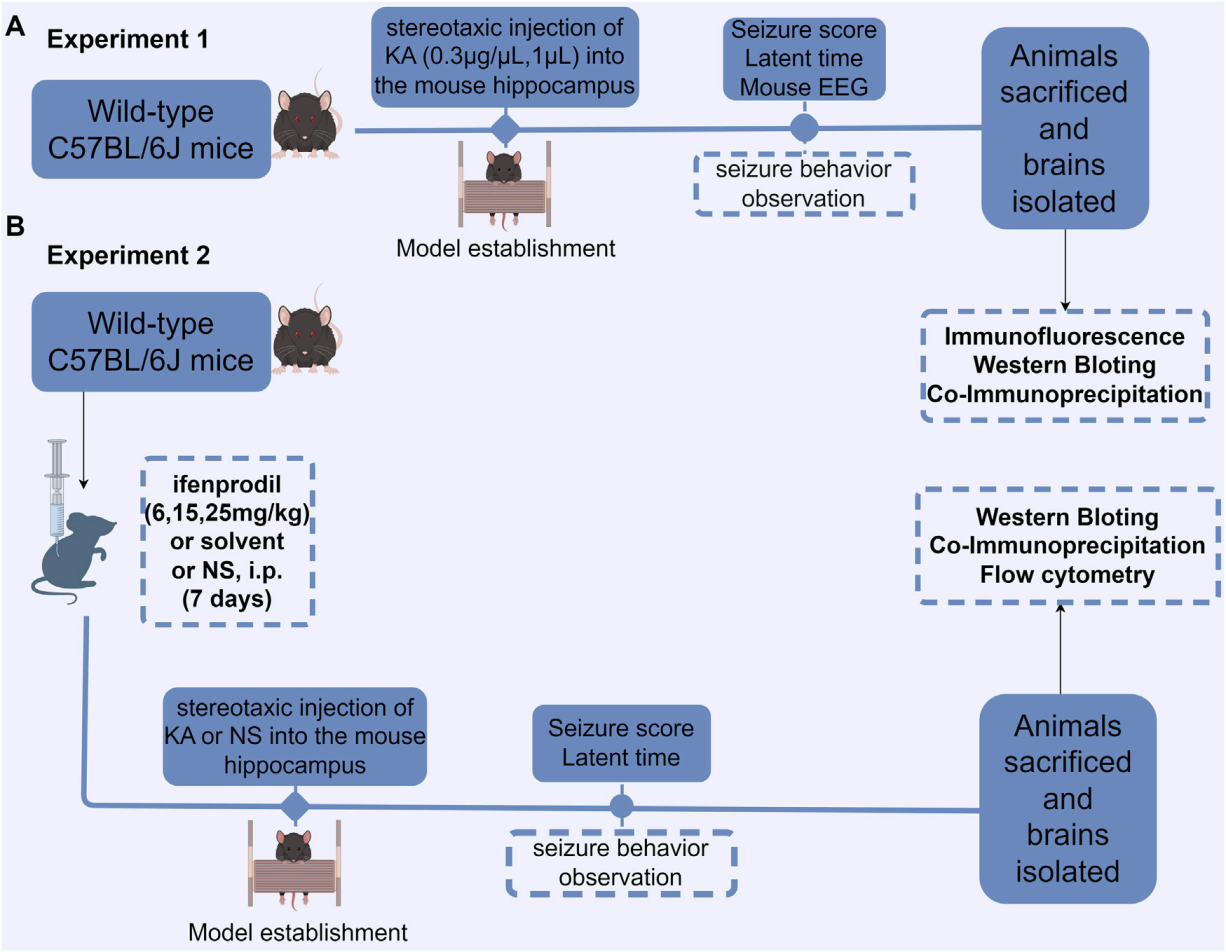
Flowchart depicting the experimental design. **(A)** Protein expression in KA-induced status epilepticus (SE) was measured. The latent period and seizure score of SE were measured and recorded following KA injection into the hippocampus, after which EEG recordings were obtained, and the brain was subsequently isolated for biochemical examination. **(B)** The effect of the GluN2B antagonist ifenprodil on KA-induced SE and its associated protein expression was evaluated. Mice were intraperitoneally injected with normal saline, ifenprodil (6, 15, 25 mg/kg), or solvent with normal saline daily for 7 consecutive days. On the seventh day, 30 min to 1 h after administration, stereotaxic injections of KA (0.3 μg/μL, 1 µL) or saline (1 µL) were administered into the hippocampus. After modeling, the mice underwent behavioral assessments, followed by brain extraction for biochemical and histological analyses. NS, normal saline; i. p., intraperitoneal injection; KA, kainic acid.

KA was administered into the hippocampus of mice to induce SE (Figure 1A). KA was dissolved in saline to create a solution with a final concentration of 0.3 μg/μL. The mice were randomly assigned to a sham operation group (n = 6) or a KA-induced SE group (n = 6). The mice in the SE group received stereotaxic injections of KA at a dose of 0.3 µg/mouse into the CA3 region of the hippocampus (coordinates: 2.75 mm, 2.46 mm, 20.63 mm) (Le Van Quyen et al., 1997; Canolty and Knight, 2010; Broggini et al., 2016; Geisler et al., 2005; Jacobson et al., 2010; Sato et al., 2017). The sham group received an intrahippocampal injection of an equivalent volume of saline. Seizures were classified as previously described (Erdoğan et al., 2005; Klaft et al., 2020). The intensity of epileptic seizures was categorized into six stages based on the Racine scale. Grade 0: absence of seizures; Grade I: whisker twitching, mastication, facial spasms; Grade II: head and face twitching characterized by nodding; Grade III: unilateral clonus with twitching of the forelimb or hindlimb; Grade IV: bilateral forelimb rhythmic twitching (tonic or tonic-clonic seizures), accompanied by an upright posture; Grade V: tonic limb twitching, dorsiflexion of the body, and falling or rolling, due to recurrent twitching. SE can be defined by any of the following criteria: 1) Grade IV or higher, sustained for 30 min; 2) Grade IV or higher; although the seizure is alleviated within 30 min, the animal fails to regain normal physical condition and consciousness; 3) Two or more Grade IV or Grade V events occurring within 30 min; 4) Continuous seizure activity lasting more than 5 min, with no return to baseline between episodes (Trinka et al., 2015; Di Nunzio et al., 2021). The latency period of seizures classified as Grade IV or higher was recorded. In this experiment, all mice injected with KA into the hippocampus developed status epilepticus (SE), resulting in a 100% success rate for the model. Additionally, no mortality was observed during the procedure. At the end of the experiment, the mice were deeply sedated with isoflurane and their hippocampi were extracted for biochemical analysis. A single animal was considered an experimental unit.

#### 2.5.2 Impact of ifenprodil on the acute seizure model

We pre-administered varying doses of the GluN2B inhibitor ifenprodil as an intervention in the KA-induced acute epilepsy model in mice (Figure 1B). Mice were randomly assigned to the sham surgery group, saline + KA group, solvent + KA group, low-dose ifenprodil + KA group, medium-dose ifenprodil + KA group, and high-dose ifenprodil + KA group (Figure 1B). Mice in the SE group received stereotactic injections of KA at a dose of 0.3 µg/mouse into the CA3 region of the hippocampus to assess the impact of ifenprodil on the KA-induced SE model. On the seventh day of the treatment regimen (as described in Section 2.4), mice received a stereotactic injection of 1 μL KA (0.3–0.4 μg/μL) into the hippocampus 0.5–1 h after administration. The sham surgery group and the model group received 1 mL of saline daily for 7 days, followed by simultaneous stereotactic injections of 1 mL of saline and 1 µL of KA (0.3–0.4 μg/μL) into the hippocampus (see Figure 1). The severity and latency of seizures classified as Grade IV or higher were recorded using the previously outlined Racine scale. The latency to epileptic seizures and latency to Racine stage IV of all the experiment groups were recorded. Following SE onset, the mice were deeply sedated with isoflurane, and their hippocampi were extracted for further biochemical analysis. A single animal was considered an experimental unit.

### 2.6 Electroencephalography (EEG) recordings

EEG recordings were conducted following KA injection and continued for over 30 min after the onset of seizure activity, and the results were independently assessed by two epilepsy specialists in a blinded manner. The mice were secured and electrodes were non-invasively attached to the forehead and temporal cortex. The Neuracle digital video EEG monitoring and analysis system (Model: Neusen. U08, Neuracle Technology, Changzhou, China) was used to capture EEG signals. Electrode placement was designated as follows: left frontal lobe (Fp1), right frontal lobe (Fp2), left temporal lobe (C3), and right temporal lobe (C4). The reference electrode (Ref) and ground wire (GND) were placed along the central axis of the mouse in the prone position. EEG signals were recorded for 30 min/mouse (Parameters: sensitivity: 10 μV/mm, paper speed: 30 mm/s, high-pass: 1 Hz, low-pass: 35 Hz, notch filter: 50 Hz). Ictal epileptic discharges, numerous spike-wave patterns, and multiple spike-and-slow-wave complexes recorded in the EEG served as benchmarks for the success of this model.

### 2.7 Western blotting

Total protein was extracted by lysing tissues in high-efficiency RIPA lysis buffer (R0010, Solarbio), containing 1% protein phosphatase inhibitor (P1260, Solarbio) and PMSF (P0100, Solarbio). Protein concentrations were measured using a BCA kit (Beyotime, P0012S). Color PAGE Gel Rapid Preparation Kit (PG110-114, Epizyme Biotech) was employed to prepare the 10% gel. The ladders (WJ103, Epizyme Biotech) were added to the gel to highlight the protein molecular weights. Additionally, 25 μg of each protein sample was separated using SDS-PAGE and transferred to a 0.2 μm PVDF membrane (ISEQ00010, Millipore) using rapid transfer buffer (WB4600, NCM Biotechnology, Suzhou, China) for 25–30 min. Following blocking with 5% BSA (SW3015, Solarbio), the membranes were incubated overnight at 4 °C with primary antibodies. The primary antibodies used were anti-phospho-CaMKII alpha antibody (1:1000, Abmart, T59748S), anti-CaMKII alpha antibody (1:1000, Abmart, T56778S), and anti-GAPDH antibody (1:1000, Abcam, ab181602). The membranes were incubated with a rabbit fluorescent secondary antibody (1:10,000, SA5-35571, Invitrogen) for 1 h at room temperature. Protein bands were detected using the Odyssey infrared imaging system (Li-COR Biosciences), and Western blot band intensity was quantified using ImageJ software (National Institutes of Health, Bethesda, MD, USA). Protein levels were quantified by normalizing to GAPDH. The experiment was repeated three times.

### 2.8 Co-immunoprecipitation

Native total protein from the hippocampus was isolated using a protein extraction kit (Invent Biotechnologies, SD-001/SN-002). The protein concentration was measured using a BCA kit (Beyotime, P0012S). An immunoprecipitation kit (Sangon Biotech, C600689-0020) was used for co-immunoprecipitation. Native total protein was incubated overnight at 4 °C with 0.5–4.0 µg of anti-GluN2B antibody per 1.0–3.0 mg of total protein lysate (Proteintech, 21920-1-AP) to form an antigen-antibody complex. PBS-rinsed Protein A/G Plus-agarose beads were incubated overnight at 4°C with the antigen-antibody complexes. The agarose beads were rinsed with IP solution, supplemented with loading buffer, heated to 95 °C for 5 min, and centrifuged to collect the immunoprecipitate. The eluted immunoprecipitates were subjected to Western blotting as described in Section 2.7. Western blot band intensity was measured using ImageJ software, and the interaction between GluN2B and CaMKII alpha was assessed using a quantitative co-immunoprecipitation approach. The GluN2B IP protein band was used to standardize the pull-down protein levels obtained via co-immunoprecipitation. The experiment was repeated three times.

### 2.9 Utilization of flow cytometry for the detection of calcium ion concentration in hippocampus cells

Following KA injection into the hippocampus to establish the SE model, the mice were deeply sedated and euthanized. The brains were promptly excised and kept cold, and the hippocampus tissues were freshly extracted. The hippocampus tissues were rinsed three times in HBSS solution (H1045, Solarbio) at 4°C. The dissected hippocampal tissue was minced and digested with five to eight times the volume of 0.125% trypsin (C100C1, NCM), and incubated at 37°C for 30 min. During digestion, the solution was removed from the incubator and gently pipetted twice. Subsequently, 2.5–3 mL of Dulbecco’s Modification of Eagle’s Medium (Vivacell, C3113) supplemented with 20% FBS (Vivacell, C04001-050X10, Shanghai, China) at 4°C was added, and digestion was stopped via pipetting. The cell pellet was filtered by passing the solution through a 200-mesh sieve (BS-70-0S, Biosharp/White Shark). The filtrate was centrifuged at 4°C and 1,000 rpm for 5 min, after which the supernatant was discarded, and the pellet was washed twice with 2.5–3 mL of HBSS solution. According to the Fluo-3AM kit guidelines (calcium ion probe Fluo-3AM, IF0150, Solarbio), 300–500 µL of 5 µM Fluo-3AM was added to the solution and incubated at 37°C for 20 min. The solution was centrifuged at 4°C and 1,500 rpm for 5 min, and the dye was discarded. The HBSS solution with 1% FBS was added in the dark and incubated at 37°C for 40 min. The cells were re-centrifuged at 4°C and 1,000 rpm for 5 min. Subsequently, the supernatant was removed, and the pellet was washed three times with HEPES buffer (H1070, Solarbio). The cells were resuspended in HEPES buffer to a concentration of 1 × 10^5^ cells/mL. The processed samples were analyzed using a flow cytometer, with an excitation wavelength of 488 nm and an emission wavelength of 526 nm. The fluorescence intensity of individual hippocampal cells was randomly measured, with each sample representing the mean fluorescence intensity of cells.

### 2.10 Statistical analysis

Continuous variables are expressed as mean ± standard deviation (SD). An independent samples t-test was used to compare the means between two groups. Additionally, one-way analysis of variance and Tukey’s honestly significant difference *post hoc* test were employed to evaluate differences among multiple groups. Statistical analysis was performed using SPSS version 23.0 software (IBM Corp., Armonk, NY). Statistical significance was set at p < 0.05.

## 3 Results

### 3.1 Behavioral observations and EEG results following KA injection into the hippocampus of mice

**FIGURE 2:**
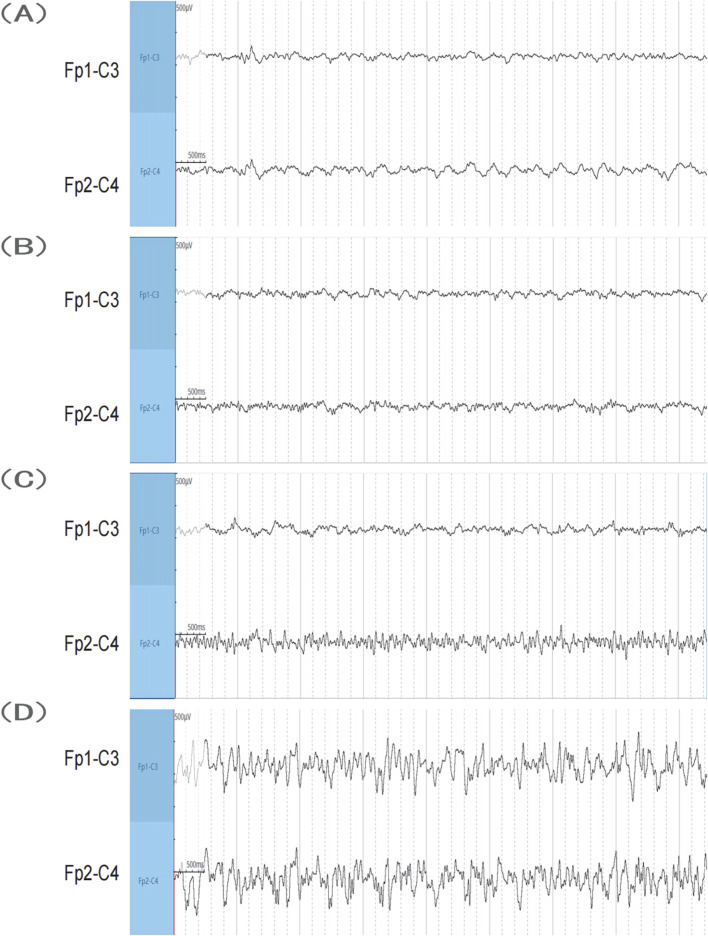
EEG results. **(A)** Sham group. **(B**,**C)** SE group before seizure after KA injection. **(D)** SE group when the seizure reached SE after KA injection. KA, kainic acid; SE, status epilepticus; EEG, electroencephalography.

In the sham operation group, wild-type C57 mice exhibited no convulsions following saline injection into the hippocampus, with normal EEG results (Figure 2A). Conversely, all mice in the SE group exhibited seizures. Following KA injection into the hippocampus, the mice exhibited grade IV or higher convulsions, successfully inducing SE in all mice. The observed seizures were partial or generalized, characterized by spinal spasms, unilateral or bilateral limb jerking, and limbic system convulsions. Some mice exhibited falls and circling behavior. Mice that reached SE were included in the study. After KA injection, behavioral changes in the mice were observed, and EEG recordings were made. No significant EEG alterations were observed during the onset latency period (Figure 2B). The onset of epileptic seizures correlated with the emergence of abnormal waveforms originating from the right side (Figure 2C). During SE, the EEG showed high-amplitude spike waves and spike-slow waves, suggesting a right-side origin, with transmission to the left and greater amplitude on the right side (Figure 2D).

### 3.2 Decreased p-CaMKIIα levels in the hippocampus of mice exhibiting KA-induced SE

**FIGURE 3:**
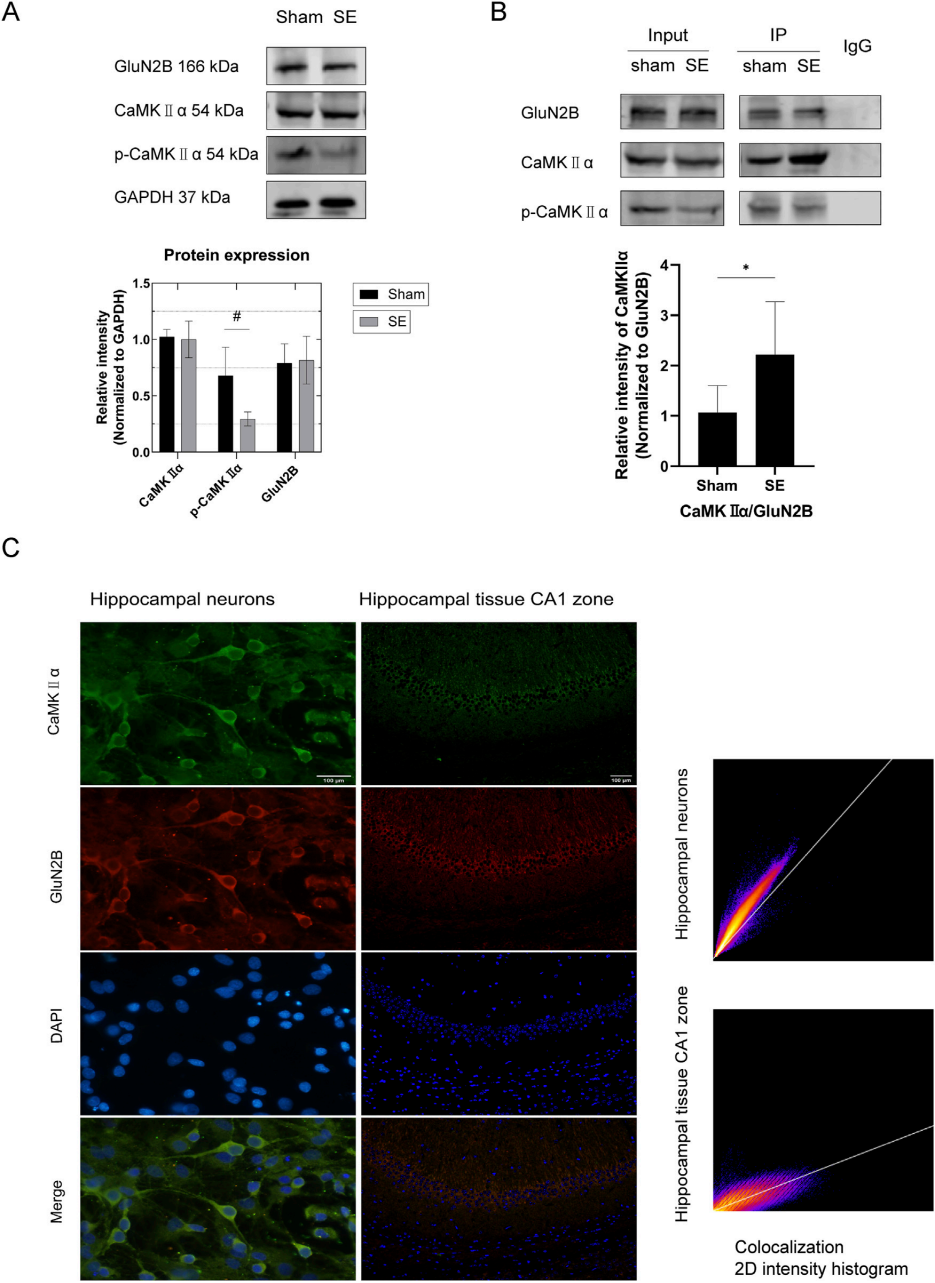
Protein expression in hippocampal tissue and interaction between GluN2B and CaMKⅡα in mouse primary neurons and hippocampal tissue. **(A)** In comparison to the sham group, the expression of p-CaMKIIα in the SE group was diminished (n = 6 per group, #p < 0.05). Nonetheless, there was no notable alteration in the expression of GluN2B and total CaMKⅡα protein. **(B)** Quantitative analysis of protein immunoprecipitation via Co-IP (*in vivo*) revealed a substantial increase in the pull-down of CaMKⅡα in the SE group compared to the control group (n = 6 per group, *p < 0.05). **(C)** Immunofluorescence colocalization examination of primary neurons (microscope: Olympus BX 53; objective lens: neuron; exposure time: 50 ms; optical/digital magnification: ×60) revealed colocalization of GluN2B and CaMKⅡα in neurons (PCC = 0.91). Scale bar: 100 µm. Immunofluorescence colocalization study of hippocampus tissue (microscope: Olympus BX 53; objective lens: hippocampal tissue; exposure time: 200 ms; optical/digital magnification: ×200) revealed that GluN2B colocalizes with CaMKⅡα in hippocampal tissue (PCC = 0.82). Scale bar: 100 µm. All trials were conducted in triplicate. The independent t-test was employed to compare mean values. KA, kainic acid; SE, status epilepticus; CaMKⅡα, calcium/calmodulin-dependent protein kinase II α subunit; DAPI, 2-(4-amidinophenyl)-6-indolecarbamidine dihydrochloride.

KA-induced seizures were observed in the experimental group, whereas no seizures were detected in the sham operation group. Mice in the experimental group were categorized according to the Racine scale, and all mice (100%) developed SE following intrahippocampal injection of KA. The protein expression levels of CaMKIIα and p-CaMKIIα in the hippocampus of mice following SE were analyzed using Western blotting. The results indicated that the total CaMKIIα protein levels in the hippocampus following SE were statistically unchanged (p > 0.05, Figure 3A); however, the phosphorylation levels of CaMKIIα were significantly reduced compared to those in the sham group (p < 0.05, Figure 3A). Collectively, our results suggest that p-CaMKIIα may play a key role in the pathophysiological process of SE.

### 3.3 Upon GluN2B binding to CaMKIIα, the phosphorylation levels of CaMKIIα decrease

To investigate the association between GluN2B and NMDARs in the hippocampus of SE mice, we utilized Western blotting to assess GluN2B expression in the hippocampus post-SE. The results showed no significant change in GluN2B expression in the hippocampus following SE (p > 0.05, Figure 3A). Immunofluorescence colocalization studies were conducted to explore the link between GluN2B and SE, as well as the interaction between GluN2B and CaMKIIα. Colocalization of GluN2B and CaMKIIα was detected in primary hippocampal neurons (Figure 3C, PCC = 0.97) and hippocampal tissue (Figure 3C, PCC = 0.82). To further confirm the interaction between GluN2B and CaMKIIα, native proteins were isolated from mouse hippocampal tissues via co-immunoprecipitation and analyzed using SDS-PAGE. The results showed that CaMKIIα was present as a pull-down protein in hippocampal tissues (Figure 3B). Quantitative co-immunoprecipitation results demonstrated a significant increase in CaMKIIα pull-down in the SE group compared to the sham group (Figure 3B, p < 0.05). Together with the findings in Section 3.2, we hypothesize that GluN2B may influence the SE process in mice by interacting with CaMKIIα and inhibiting its phosphorylation.

### 3.4 GluN2B in the hippocampus of SE mice inhibits CaMKIIα phosphorylation, disrupting calcium ion homeostasis and contributing to epileptic convulsions

#### 3.4.1 Inhibitory effect of ifenprodil, a GluN2B antagonist, on acute epileptic convulsions induced by KA

**FIGURE 4:**
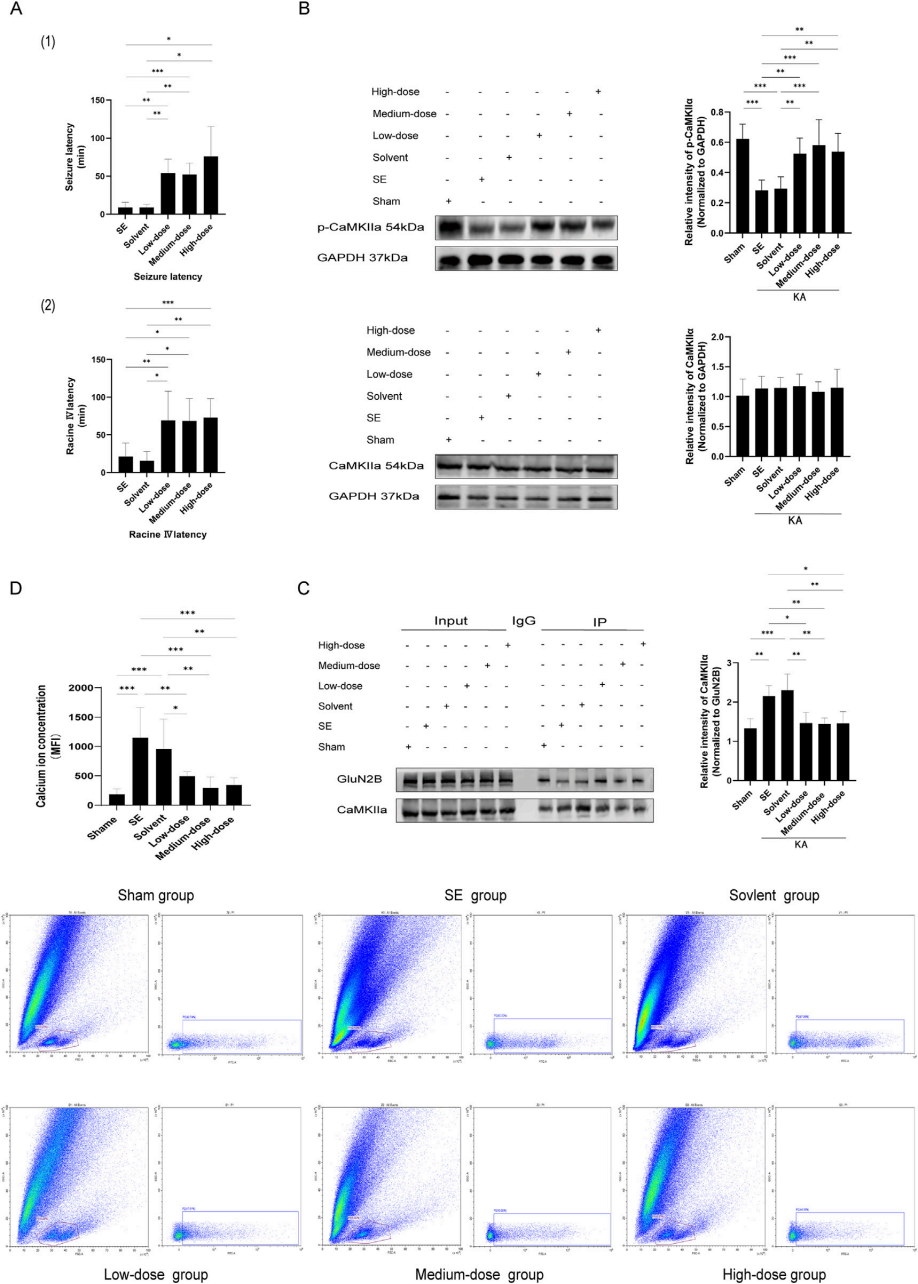
The GluN2B inhibitor ifenprodil impedes GluN2B, alters the interaction between GluN2B and CaMKⅡα, diminishes the occupancy of CaMKⅡα phosphorylation sites, affects the phosphorylation level of CaMKⅡα, disrupts intracellular calcium ion homeostasis, and decreases both epileptic sensitivity and the severity of KA-induced status epilepticus. **(A)** The graphs illustrate (1) the latency of seizure onset and (2) the latency from seizure to Racine IV. In comparison to the SE group, the latency of seizure onset and the latency to Racine IV in the ifenprodil intervention group were significantly prolonged (n = 7 in each group, **p < 0.01, ***p < 0.001), with no substantial discrepancies observed among the various dose groups (p > 0.05). **(B)** In comparison to the sham operation group, p-CaMKⅡα levels in the SE group and the solvent group were markedly reduced (n = 7 in each group, **p < 0.01, ***p < 0.001). Following ifenprodil intervention, p-CaMKⅡα levels increased relative to the SE and solvent groups, with no substantial discrepancies observed among the various doses of ifenprodil (p > 0.05). Additionally, no substantial discrepancies in total CaMKⅡα protein levels were observed across the different groups (n = 5 in each group, p > 0.05). **(C)** In comparison to the sham operation group, the pull-down of CaMKⅡα in SE and solvent groups was significantly increased (n = 5 in each group, *p < 0.05, **p < 0.01, ***p < 0.001). However, following ifenprodil intervention, CaMKⅡα levels were significantly diminished relative to the SE and solvent groups, with no notable differences observed among the groups receiving varying doses of ifenprodil intervention (p > 0.05). **(D)** Compared to the sham operation group, the calcium ion concentration in the hippocampal tissue cells of the SE group and the solvent group was markedly elevated (n = 5 in each group, **p < 0.01, ***p < 0.001). Conversely, following ifenprodil intervention, the calcium ion concentration in the hippocampal tissue cells significantly decreased relative to the SE group and the solvent group (n = 5 in each group, *p < 0.05, **p < 0.01, ***p < 0.001), with no significant differences observed among the groups receiving varying doses of ifenprodil (p > 0.05). KA, kainic acid; SE: status epilepticus; CaMKⅡα: calcium/calmodulin-dependent protein kinase II α subunit.

The groups included the sham operation group (NS + NS), the model group (NS + KA), the solvent group (PBS+6% DMSO + KA), the low-dose group (6 mg/kg ifenprodil + KA), the medium-dose group (15 mg/kg ifenprodil + KA), and the high-dose group (25 mg/kg ifenprodil + KA). No epileptic seizures were observed in the sham operation group. Compared to the model group and solvent group, the latency to epileptic seizures and latency to Racine stage IV in the ifenprodil intervention groups were significantly extended (Figure 4A, p < 0.05). However, no significant differences were observed among the low-, medium-, and high-dose groups (p > 0.05). This suggests that ifenprodil exerts a strong inhibitory effect on KA-induced acute epileptic convulsions.

#### 3.4.2 Ifenprodil intervention suppresses the interaction between GluN2B and CaMKIIα in the hippocampus of SE mice and elevates CaMKIIα phosphorylation levels

The models were established after 7 days of ifenprodil pretreatment. Compared to the sham operation group, p-CaMKIIα levels in the model group and solvent group were significantly reduced (Figure 4B, p < 0.05). However, after ifenprodil intervention, p-CaMKIIα levels significantly increased (Figure 4B, p < 0.05). Simultaneously, native proteins were isolated from the hippocampal tissue of mice for SDS-PAGE using the co-immunoprecipitation technique. The results showed that CaMKIIα was present as a pulled-down protein in the hippocampal tissue (Figure 4C). Quantitative protein immunoprecipitation results indicated that, compared to the control group, the pull-down of CaMKIIα in both the model group and solvent group was significantly increased (Figure 4C, p < 0.05), while CaMKIIα levels significantly decreased following ifenprodil intervention (Figure 4C, p < 0.05). Our findings suggest that GluN2B in the hippocampus of SE mice influences the onset and progression of epilepsy by interacting with CaMKIIα and suppressing its phosphorylation.

#### 3.4.3 Ifenprodil reduces calcium ion levels in hippocampal cells of SE mice

The CaMKⅡα subunit plays a critical role in Ca^2+^ transport across various neuronal types and can bind to Ca^2+^ to form a Ca^2+^/CaM (calmodulin) complex (Yamagata et al., 2006). Therefore, we employed flow cytometry to quantify intracellular calcium ion levels. The results showed that, compared to the sham operation group, calcium ion concentrations in the hippocampal cells of mice in both the model group and solvent group were significantly elevated following KA-induced SE (Figure 4D, p < 0.05). In contrast, the calcium ion concentration in hippocampal cells of SE mice treated with the GluN2B inhibitor ifenprodil was significantly reduced (Figure 4D, p < 0.05).

## 4 Discussion

### 4.1 Development of SE is linked to the phosphorylation status of CaMKⅡα

CaMKII is a versatile protein kinase predominantly found in the brain, where it modulates neuronal activity through various mechanisms (Yamagata et al., 2006). Alterations in the phosphorylation state of CaMKII subunits can affect the enzyme’s kinetic properties, regulate its translocation, and impact its activity. Numerous studies have reported changes in CaMKII phosphorylation, indicating altered enzymatic activity. Specifically, a higher ratio of autophosphorylated (Thr 286 site) to non-autophosphorylated CaMKII correlates with increased baseline activity of the enzyme (Weeber et al., 2003). Increasing evidence suggests that CaMKIIα plays a pivotal role in epileptic seizures. Altered CaMKII function has been observed in various epileptic seizure models and other forms of heightened neuronal excitability. Elevated neuronal excitability and epilepsy may be associated with reduced and impaired CaMKII activity (Churn et al., 2000). Moreover, the inactivation of CaMKII during epileptic seizures may affect neuronal survival after the seizure. Increasing CaMKII phosphorylation in the hippocampus may prevent severe syncope in epileptic rats (Zirotti Rosenberg et al., 2022). Consistent with previous research, our findings demonstrated a notable reduction in p-CaMKIIα in the hippocampus of KA-induced SE mice.

### 4.2 GluN2B binds to CaMKIIα, occupying its phosphorylation site and thereby reducing CaMKIIα phosphorylation levels

In our study, the concentration of p-CaMKIIα decreased in the hippocampus of SE mice. We focused on the mechanisms leading to reduced CaMKIIα phosphorylation levels in SE. When the intracellular calcium ion concentration rises, CaMKII can be activated by the binding of calcium ions and calmodulin. After activation, CaMKII undergoes autophosphorylation at the Thr286 site, thereby generating spontaneous activity (Tullis et al., 2023). However, our experimental results and those of previous studies (Blair et al., 2008) indicate that the calcium ion level in cells after epilepsy is elevated, while the level of p-CaMKIIα is decreased and its activity is reduced. This is inconsistent with our experimental results. Therefore, we consider that the decrease in p-CaMKIIα level during status epilepticus is not dependent on the calcium ions/calmodulin pathway. Previous research suggests NMDARs are critical for excitatory neurotransmission, and hyperactivity of GluN2B-containing NMDARs may be associated with the onset of epilepsy. (Chen et al., 2020; Myers et al., 2019). Our findings showed no significant change in the total protein expression of GluN2B in the hippocampus of SE mice compared to the sham group. Previous research has shown that CaMKII can associate and interact with the NMDAR subunit GluN2B (O'Leary et al., 2011). The regulated binding of CaMKII to GluN2B promotes Ca^2+^/CaM-induced translocation of CaMKII to neuronal synapses (Bayer et al., 2006). Additionally, GluN2B is the primary binding partner of CaMKII. Their interaction regulates CaMKII activity and is critical for maintaining synaptic strength, synaptic plasticity, and memory (Yasuda et al., 2022; Barcomb et al., 2016; Jalan-Sakrikar et al., 2012). Consequently, we hypothesized that the interaction between GluN2B and CaMKIIα affects CaMKIIα phosphorylation levels. To investigate the relationship between CaMKIIα and GluN2B, we performed immunofluorescence colocalization analysis, which revealed that CaMKIIα and GluN2B colocalized in primary neurons and hippocampal tissue. This result is consistent with previous research (Barria and Malinow, 2005; Halt et al., 2012). Simultaneously, to investigate the regulatory mechanism of CaMKIIα and GluN2B in SE, we used co-immunoprecipitation analysis, which showed that CaMKIIα associates with GluN2B to form a protein complex. The interaction between CaMKIIα and GluN2B increased, while p-CaMKIIα levels decreased in the KA-induced SE model. Mutation studies have shown that active CaMKII associates with the Ser 1303 site on GluN2B via its catalytic domain, while the secondary binding site on GluN2B requires prior autophosphorylation of CaMKII at Thr286 (O'Leary et al., 2011). Moreover, GluN2B binds to the T site of CaMKII and regulates its catalytic activity (Cheriyan et al., 2011). Previous *in vitro* studies have shown that CaMKII forms a prolonged association with GluN2B at the T286 binding site (“T site”), maintaining the displacement of the autoregulatory domain and enabling Ca^2+^/CaM-independent kinase activity. The binding site of the peptide inhibitor of CaMKIIα is also the binding site of GluN2B(Tullis et al., 2023). Following the binding of CaMKIIα to Ca^2+^/calmodulin (CaM), CaMKIIα exposes a surface depression containing three docking sites, including a substrate-binding pocket and a region where Thr286 is embedded, which is capable of binding GluN2B, as well as some substrates and inhibitors (Nicoll and Schulman, 2023). Thus, CaMKIIα and GluN2B may interact and regulate activity at the phosphorylation site of CaMKIIα (p-T286). We hypothesized that the CaMKIIα-GluN2B protein complex increased in KA-induced SE animals, occupying the CaMKIIα phosphorylation site and leading to a reduction in CaMKIIα phosphorylation levels.

### 4.3 Inhibiting GluN2B can elevate the phosphorylation levels of CaMKIIα, which may improve the condition of SE

Following the administration of the GluN2B inhibitor ifenprodil, our hypothesis was confirmed. GluN2B inhibition resulted in increased phosphorylation of CaMKIIα in SE mice. The CaMKIIα and GluN2B protein complexes were significantly reduced, delaying the onset of epilepsy. This observation aligns with previous research. Ifenprodil is the first neuroprotective agent identified as selective for NMDARs containing GluN2B subunits (Carter et al., 1989; Kryukov et al., 2016). The pharmacological inhibition of NMDARs using GluN2B-specific antagonists immediately after SE may offer neuroprotection without impairing cognitive function or memory (Kryukov et al., 2016). Ifenprodil acts as a selective antagonist of NMDARs containing the GluN2B subunit and exhibits anticonvulsant properties (Abbasova et al., 2018). As an allosteric inhibitor, it binds to the bilobed structure of the GluN1-GluN2B heterodimer, thereby altering the structure and function of GluN2B (Schreiber et al., 2019; Tajima et al., 2016). The inhibition of GluN2B-containing receptors or CaMKII hinders long-term potentiation. Strack et al. developed a GluN2B construct with a slight mutation in its cytoplasmic tail, which led to a marked reduction in its binding to active CaMKII in *in vitro* assays (Strack et al., 2000).

### 4.4 GluN2B inhibitors reduce the number of CaMKIIα phosphorylation sites occupied by GluN2B, increase CaMKIIα phosphorylation levels, and restore calcium ion homeostasis

Our experimental findings indicated that the administration of the GluN2B inhibitor ifenprodil mitigated the reduction of p-CaMKIIα in SE mice, counteracted the increase of the CaMKIIα-GluN2B protein complex, restored intracellular calcium ion levels, and corrected dysregulated calcium homeostasis. Alterations in calcium homeostasis have been observed during the initiation and maintenance of epileptic convulsions (Pal et al., 1999) and depend on the NMDAR pathway (DeLorenzo et al., 1998; Myers et al., 2019). Additionally, elevated intracellular calcium levels may inhibit the activation of CaMKII (Avalos-Fuentes et al., 2015). Research indicates that TRPV4 mutations can elevate intracellular Ca^2+^ via a CaMKII-mediated pathway and that inhibiting CaMKII can prevent the increase in intracellular Ca^2+^ and neurotoxicity in cultured primary mouse neurons (Woolums et al., 2020). Ashpol et al. found that using small molecule and peptide inhibitors to block CaMKII-induced apoptosis in cultured cortical neurons disrupts neuronal calcium signaling and glutamate homeostasis, increases neuronal excitotoxicity, and exacerbates programmed cell death (Ashpole et al., 2012). These results align with our experimental findings. Consequently, our results suggest that the binding of GluN2B to CaMKIIα may be inhibited by the GluN2B-specific antagonist ifenprodil, leading to increased phosphorylation of CaMKIIα and affecting intracellular calcium homeostasis. Ifenprodil may directly influence calcium channels, modulate calcium influx, regulate changes in the intracellular calcium buffer calmodulin (Avalos-Fuentes et al., 2015), and affect CaMKIIα activity. This results in changes in calcium homeostasis, neuronal excitability, and the E/I balance in the central nervous system, ultimately affecting the onset and progression of SE.

However, our study has several limitations. The precise mechanism linking reduced phosphorylation of CaMKIIα to the imbalance in calcium homeostasis remains unclear. Further research is needed to clarify the exact mechanism that GluN2B binding reduces CaMKIIα phosphorylation.

## 5 Conclusion

The reduction in the phosphorylation level of CaMKⅡα in the hippocampus of SE mice depends on the NMDAR pathway. The proposed mechanism involves GluN2B binding to CaMKⅡα, thereby obstructing its phosphorylation site, which inhibits CaMKⅡα phosphorylation and subsequently reduces its phosphorylation level and activity. Additionally, disruption of NMDAR-dependent calcium homeostasis impairs the E/I balance in the central nervous system, thereby affecting the onset and progression of epilepsy. These findings highlight a novel target and potential treatment strategies to mitigate epileptic seizures and prevent SE.

## Data Availability

The original contributions presented in the study are included in the article/supplementary material, further inquiries can be directed to the corresponding author.

## Abbreviations

KA, kainic acid; NMDAR, N-methyl-D-aspartate receptor; PCC, Pearson correlation coefficient; SE, Status epilepticus; DMSO, dimethyl sulfoxide; CaMKII, calcium/calmodulin-dependent protein kinase II; E/I, excitation/inhibition; PBS, phosphate-buffered saline.
